# Metabolic consequences of HIF silencing in a triple negative human breast cancer xenograft

**DOI:** 10.18632/oncotarget.24569

**Published:** 2018-02-24

**Authors:** Santosh K. Bharti, Yelena Mironchik, Flonne Wildes, Marie-France Penet, Eibhlin Goggins, Balaji Krishnamachary, Zaver M. Bhujwalla

**Affiliations:** ^1^ Division of Cancer Imaging Research, The Russell H. Morgan Department of Radiology and Radiological Science, School of Medicine, The Johns Hopkins University, Baltimore, MD, USA; ^2^ Sidney Kimmel Comprehensive Cancer Center, School of Medicine, The Johns Hopkins University, Baltimore, MD, USA; ^3^ Department of Radiation Oncology and Molecular Radiation Sciences, School of Medicine, The Johns Hopkins University, Baltimore, MD, USA

**Keywords:** breast cancer xenografts, hypoxia inducible factor, hypoxia, MR spectroscopy, metabolism

## Abstract

Hypoxia is frequently encountered in tumors and results in the stabilization of hypoxia inducible factors (HIFs). These factors transcriptionally activate genes that allow cells to adapt to hypoxia. In cancers, hypoxia and HIFs have been associated with increased invasion, metastasis, and resistance to chemo and radiation therapy. Here we have characterized the metabolic consequences of silencing HIF-1α and HIF-2α singly or combined in MDA-MB-231 triple negative human breast cancer xenografts, using non-invasive proton magnetic resonance spectroscopic imaging (^1^H MRSI) of *in vivo* tumors, and high-resolution ^1^H MRS of tumor extracts. Tumors from all three sublines showed a significant reduction of growth rate. We identified new metabolic targets of HIF, and demonstrated the divergent consequences of silencing HIF-1α and HIF-2α individually on some of these targets. These data expand our understanding of the metabolic pathways regulated by HIFs that may provide new insights into the adaptive metabolic response of cancer cells to hypoxia. Such insights may lead to novel metabolism based therapeutic targets for triple negative breast cancer.

## INTRODUCTION

Hypoxic tumor microenvironments are frequently detected in cancers and contribute to aggressiveness [1], resistance to treatment [2], and metastatic dissemination [3, 4]. The adaptive response of cancer cells to hypoxia is mediated through the stabilization of hypoxia inducible factors (HIFs) that increase the transcription of several genes, by binding to hypoxia response elements (HREs) in the promoter region of these genes [5]. HIF is a heterodimeric basic helix-loop-helix PAS (Per-ARNT-Sim) domain containing transcription factor that consists of one of three oxygen-regulated α-subunits, HIF-1α, HIF-2α and HIF-3α and a constitutively expressed β-subunit (HIF-β/ARNT) [6, 7]. The α-subunits are constitutively transcribed and translated, but are regulated at the protein level by oxygen-dependent hydroxylation of specific prolyl residues, and degraded due to the presence of an oxygen–dependent degradation domain (ODD) [8]. Most target genes activated in response to hypoxia have been identified through their regulation by HIF-1α [5]. However, recent studies have identified target genes that are regulated by HIF-2α or by the overlapping activity of both HIF-1α and HIF-2α [9].

Triple negative breast cancers (TNBCs) are associated with a higher rate of recurrence than hormone responsive cancers [10, 11]. In TNBCs, overexpression of HIF-1α was associated with poor outcome in early stage disease [12]. Our purpose here was to expand our understanding of the consequences of hypoxia and HIFs on tumor metabolism using *in vivo* ^1^H magnetic resonance spectroscopic imaging (MRSI) and *ex vivo* high resolution ^1^H MR spectroscopy (MRS) of TNBC xenografts. Such insights can identify novel metabolic targets for TNBC treatment and expand our understanding of the role of hypoxia and HIF in creating a more aggressive phenotype.

Most studies characterizing the consequences of HIF-1α and HIF-2α silencing on metabolism have been performed with cancer cells but, to the best of our knowledge, none with breast cancer cells [13, 14]. We recently developed MDA-MB-231 cells with HIF-1α (sh-HIF-1α), HIF-2α (sh-HIF-2α), and both HIF-1α and HIF-2α silenced (sh-HIF-1/2α) [15]. Cells in culture showed significant differences in lipids, glucose consumption and lactate production with HIF silencing [15]. Here, for the first time, we have characterized the metabolism of tumors derived from these triple negative MDA-MB-231 breast cancer cells *in vivo* and *ex vivo*. Using non-invasive ^1^H MRSI of orthotopically implanted tumors we detected significant changes in total choline and lipid signals *in vivo*. High-resolution ^1^H MRS of tumor extracts identified significant changes in some branched chain amino acids, organic acids, choline metabolites, substrates, and nucleotides in the aqueous phase, and triglycerides and lipids in the lipid phase of sh-HIF-1α, sh-HIF-2α and sh-HIF-1/2α tumors. While there was some commonality of the changes across the sublines, in some instances the directionality of the changes was opposite between HIF-1α and HIF-2α silencing. Tumors from all three sublines showed a significant reduction of growth rate. Metabolic changes identified in these tumors may reveal effective targets in TNBC treatment.

## RESULTS

### Tumor growth curves and validation of HIF-silencing

**Figure 1:**
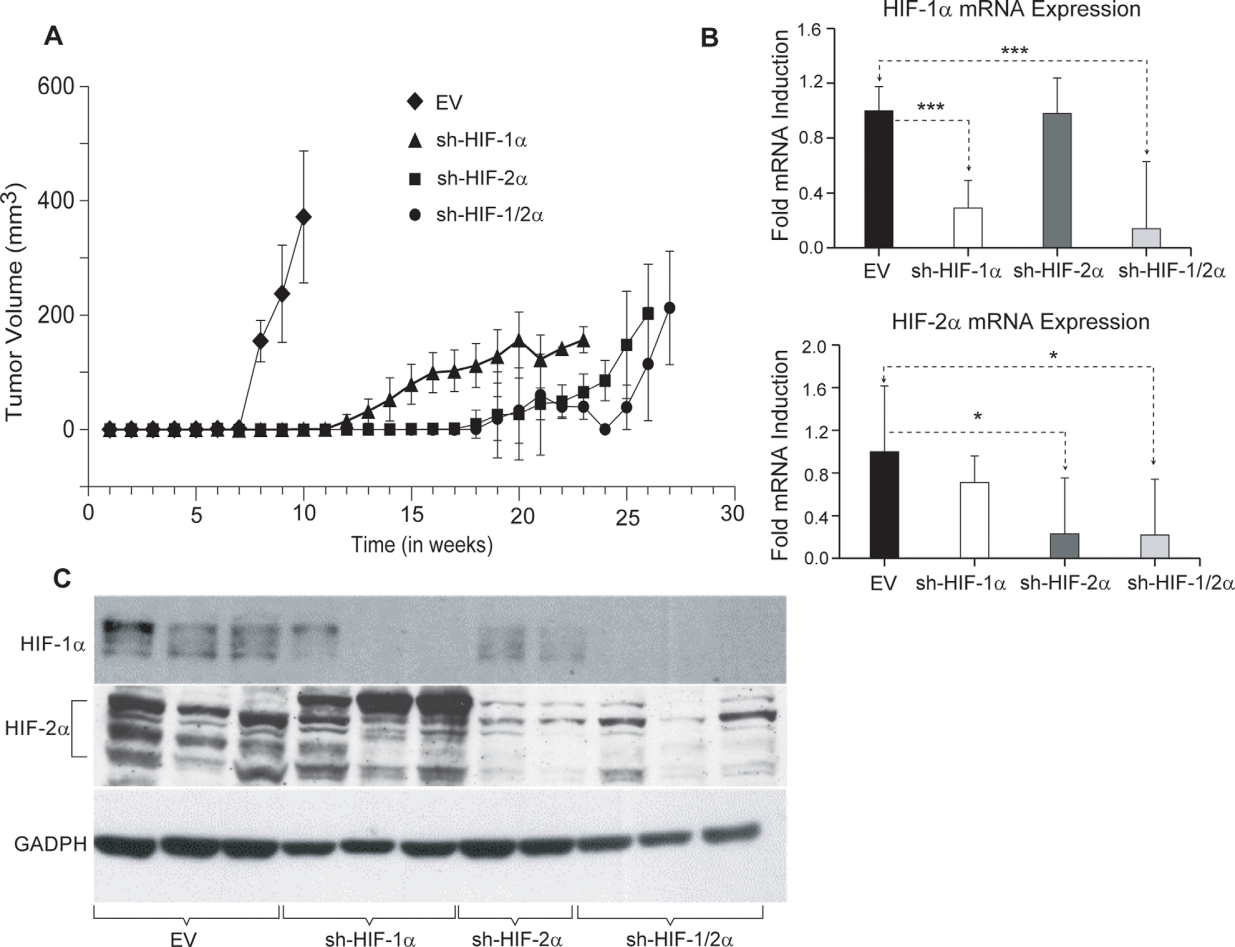
(**A**) Growth characteristics of tumor xenografts derived from genetically engineered 231-EV (*n* = 9, diamond), sh-HIF-1α (*n* = 10, triangle), sh-HIF-2α (*n* = 9, square) and sh-HIF-1/2α (*n* = 7, dot) cells. Growth curves were generated by averaging the tumor volume of mice in each group. (**B**) RT q-PCR analysis showing HIF-1α and HIF-2α mRNA expression levels in 231-EV (*n* = 8), sh-HIF-1α (*n* = 10), sh-HIF-2α (7-8) and sh-HIF-1/2α (*n* = 5) tumors. Values represents Mean fold induction ± SEM of the ∆Ct Values. *P*-values less than 0.05 were considered significant. ^*^p ≤ 0.05, ^***^ *p* ≤ 0.0005. (**C**) Representative immunoblots showing HIF-1α and HIF-2α levels in 231-EV, sh-HIF-1α, sh-HIF-2α and sh-HIF-1/2α tumors. GAPDH was used as a loading control in the western blots.

To determine the effect of HIF down-regulation on tumor growth, tumor volumes were measured weekly following inoculation of cells in the mammary fat pad of female SCID mice. As shown in Figure 1A, compared to 231-EV tumors (empty vector MDA-MB-231 with no shRNA), sublines expressing shRNA downregulating HIF-1α, HIF-2α or both isoforms of HIF showed delayed onset of tumor growth as well as growth rate. Excised tumors analyzed for mRNA in Figure 1B exhibited a significant decrease of HIF-1α and HIF-2α mRNA expression or both in the corresponding silenced tumors. Western blot analysis of the tumors confirmed changes in HIF protein levels in tumors derived from the corresponding cells lines as shown in Figure 1C.

### HIF silencing alters choline and lipid metabolism *in vivo*

**Figure 2:**
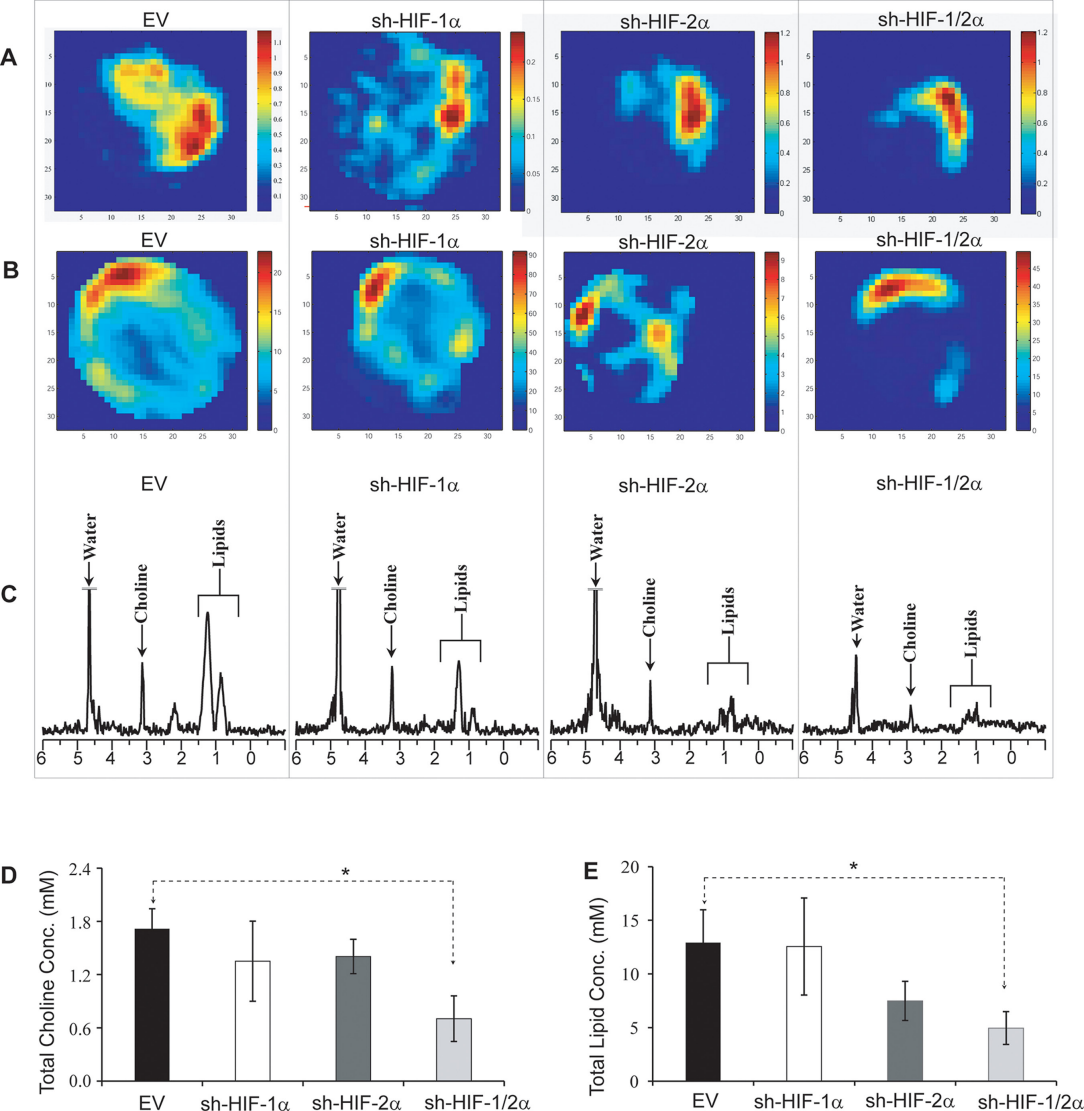
Representative (**A**) total choline and (**B**) lipid maps of tumors from 231-EV, sh-HIF-1α, sh-HIF-2α and sh-HIF-1/2α tumors. (**C**) Corresponding *in vivo* MR spectra showing choline and lipid signals at 3.2 and 1.0 ppm respectively. Bar plot showing quantitative analysis of (**D**) total choline and (**E**) lipids. Values represent mean ± SEM. ^*^p ≤ 0.05. The total choline signal consists of free choline, phosphocholine and glycerophosphocholine. The lipid signal may contain signal from lactate.

To determine if HIF downregulation altered choline and lipid metabolism, we next performed *in vivo* ^1^H MRSI of orthotopic tumors derived from 231-EV and HIF silenced sublines. As shown in the representative total choline and lipid maps in Figure 2A and 2B, a decrease in total choline and lipids was observed with HIF silencing, especially when both HIF-1α and HIF-2α were silenced. Spectra obtained from the corresponding 4 mm thick slice are displayed in Figure 2C. Quantitative analyses of *in vivo* total choline and total lipid data acquired from multiple tumors are summarized in Figure 2D and 2E. Compared to the empty vector control tumors, a significant decrease in both total choline and lipid levels was observed in tumors when both HIF-1α and HIF-2α were silenced. Also evident is the spatial heterogeneity of total choline and lipids in these tumors.

### Metabolic profile of tumor extracts

**Figure 3:**
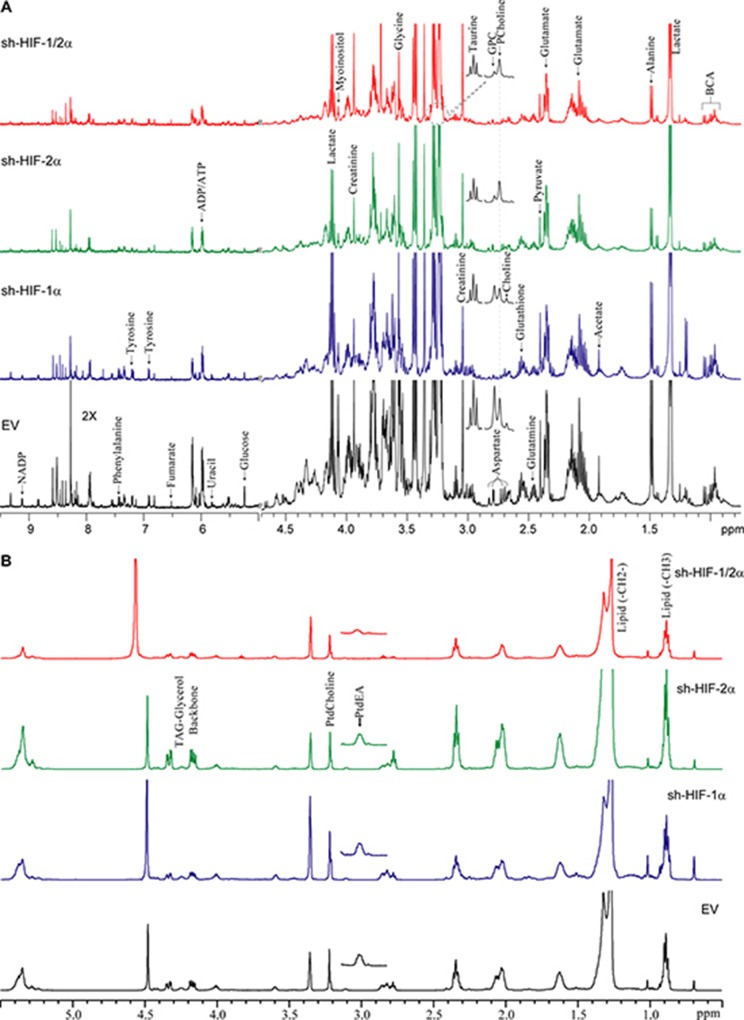
(**A**) Representative high-resolution ^1^H MR spectra obtained from the aqueous phase of 231-EV, sh-HIF-1α, sh-HIF-2α shRNA and sh-HIF-1/2α tumors. GPC: glycerophosphocholine, PCholine: phosphocholine, BCA: branched chain amino acids. (**B**) Representative high-resolution ^1^H MR spectra obtained from the lipid phase of 231-EV, sh-HIF-1α, sh-HIF-2α and sh-HIF-1/2α tumors. All spectra were plotted on the same vertical scale and acquired with identical experimental parameters. PtdEA: phosphatidylethanolamine, PtdCholine: phosphatidylcholine.

**Figure 4:**
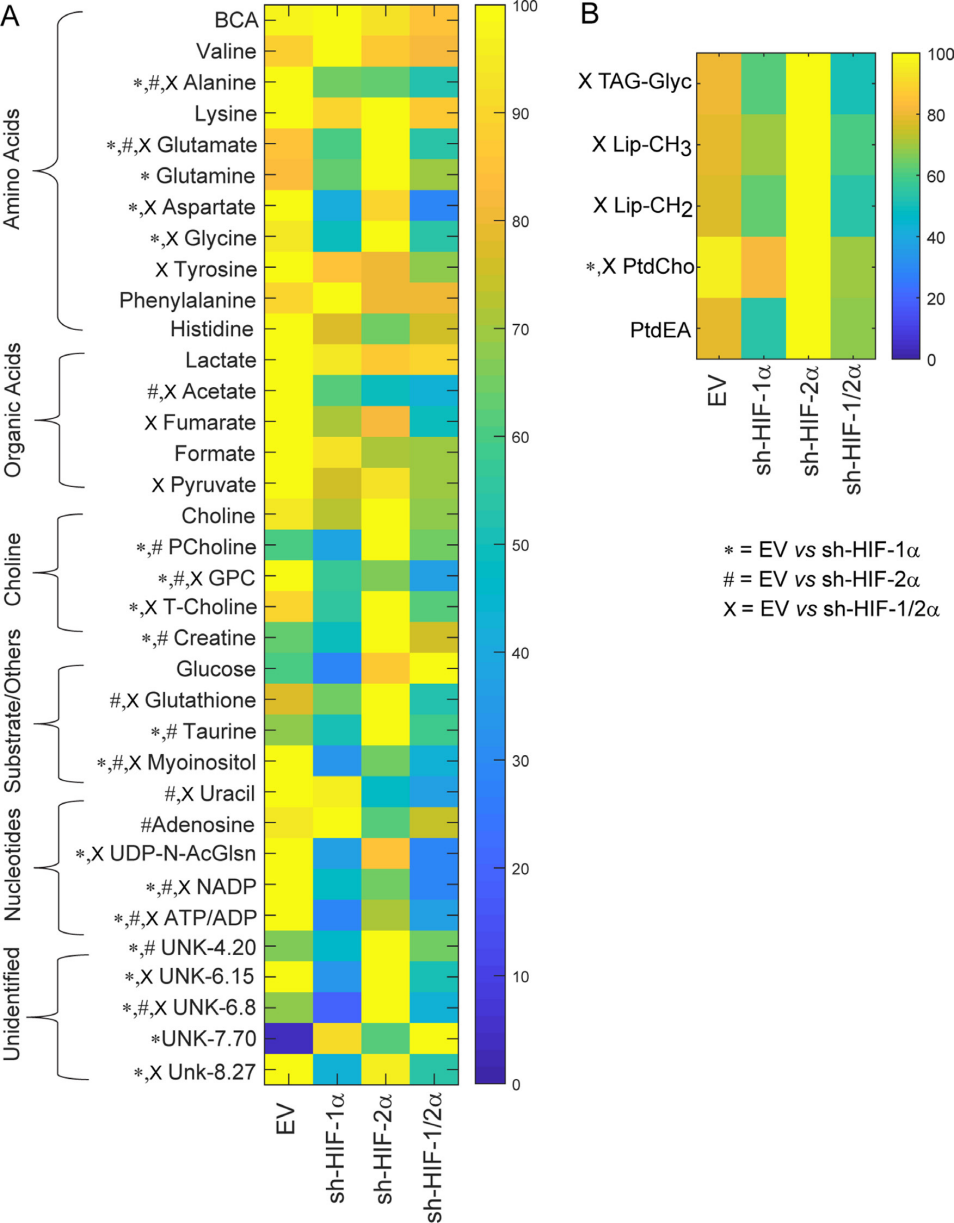
Metabolic heat maps representing the differences in the metabolic profile of tumors Heat maps were generated from quantitative analysis of high-resolution one dimensional ^1^H MR spectral data from (**A**) the aqueous phase and (**B**) the lipid phase of 231-EV, sh-HIF-1α, sh-HIF-2α and sh-HIF-1/2α tumor extracts. Heat maps were created using MATLAB software (MATLAB R2012b, MathWorks) to visualize the metabolic patterns. Due to the high dynamic range of metabolites, we normalized the highest intensity of a metabolite in each of the four groups to 100%. This normalization provides a dynamic range between 0 - 100%, allowing a better presentation of heat maps. The heat map represents average measurements of multiple replicates per group (231-EV: *n* = 9, sh-HIF-1α: *n* = 10, sh-HIF-2α: *n* = 9, and sh-HIF-1/2α: *n* = 7). The integral area under the peak was normalized to weight and volume of the sample. TSP dissolved in D_2_O was used as a quantitative reference in the spectral analysis. Unpaired student’s *t*-test was applied to measure statistical significance. (^*^p value < 0.05 for 231-EV *vs* sh-HIF-1α; ^#^*p* value < 0.05 for 231-EV *vs* sh-HIF-2α; ^x^*p* value ≤ 0.05 for 231-EV *vs* sh-HIF-1/2α). UDP-N-AcGlsn: UDP-N-Acetyl-Glucosamine.

We performed high-resolution ^1^H MRS of the aqueous and lipid phases of tumor extracts to expand the characterization of metabolites in these tumors. As shown in representative aqueous phase spectra in Figure 3A and lipid phase spectra in Figure 3B, differences in tumor metabolic patterns in both aqueous and lipid phase spectra were detected in sh-HIF-1α, sh-HIF-2α and sh-HIF-1/2α tumors compared to 231-EV tumors. Heat maps were derived from the quantitative values of the different signals detected in the spectra to provide an overview of the changes in amino acids, organic acids, choline compounds, substrates/other, nucleotides, as well as some unidentified signals from the aqueous phase (Figure 4A) and signals detected from the lipid phase (Figure 4B). Of the amino acids detected in the spectra, HIF downregulation significantly altered alanine, glutamate, glutamine, aspartate, glycine, and tyrosine. Acetate, fumarate and pyruvate, but not lactate, were the organic acids that were significantly altered by HIF silencing. Consistent with the *in vivo* data, phosphocholine, glycerophosphocholine and total choline were significantly altered with HIF silencing. Consistent with the absence of changes in lactate, there were no changes in glucose levels, but creatine, glutathione, taurine and myoinositol significantly changed with HIF silencing. Signals from nucleotides such as UDP-N-AcGlsn (UDP-N-Acetyl Glucosamine), NADP, and ATP were also significantly altered. In the lipid phase spectra, signals from TAG-Glyc, lipids (CH_3_ and CH_2_), and from phosphatidylcholine but not phosphatidylethanolamine were significantly altered.

**Figure 5:**
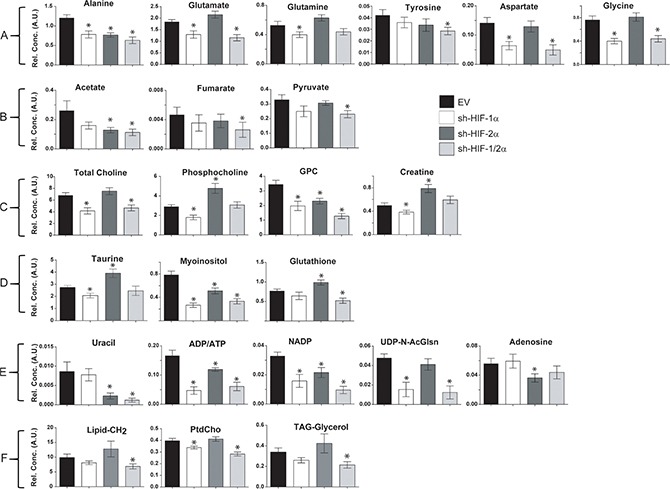
Bar plots of significantly different concentrations in arbitrary units (A.U.) detected in (**A**) amino acids, (**B**) organic acids, (**C**) choline metabolites, (**D**) substrates/other, (**E**) nucleotides, and (**F**) lipids in 231-EV, sh-HIF-1α, sh-HIF-2α and sh-HIF-1/2α tumors. Values represent mean ± SEM. ^*^p value ≤ 0.05 compared to 231-EV.

**Figure 6:**
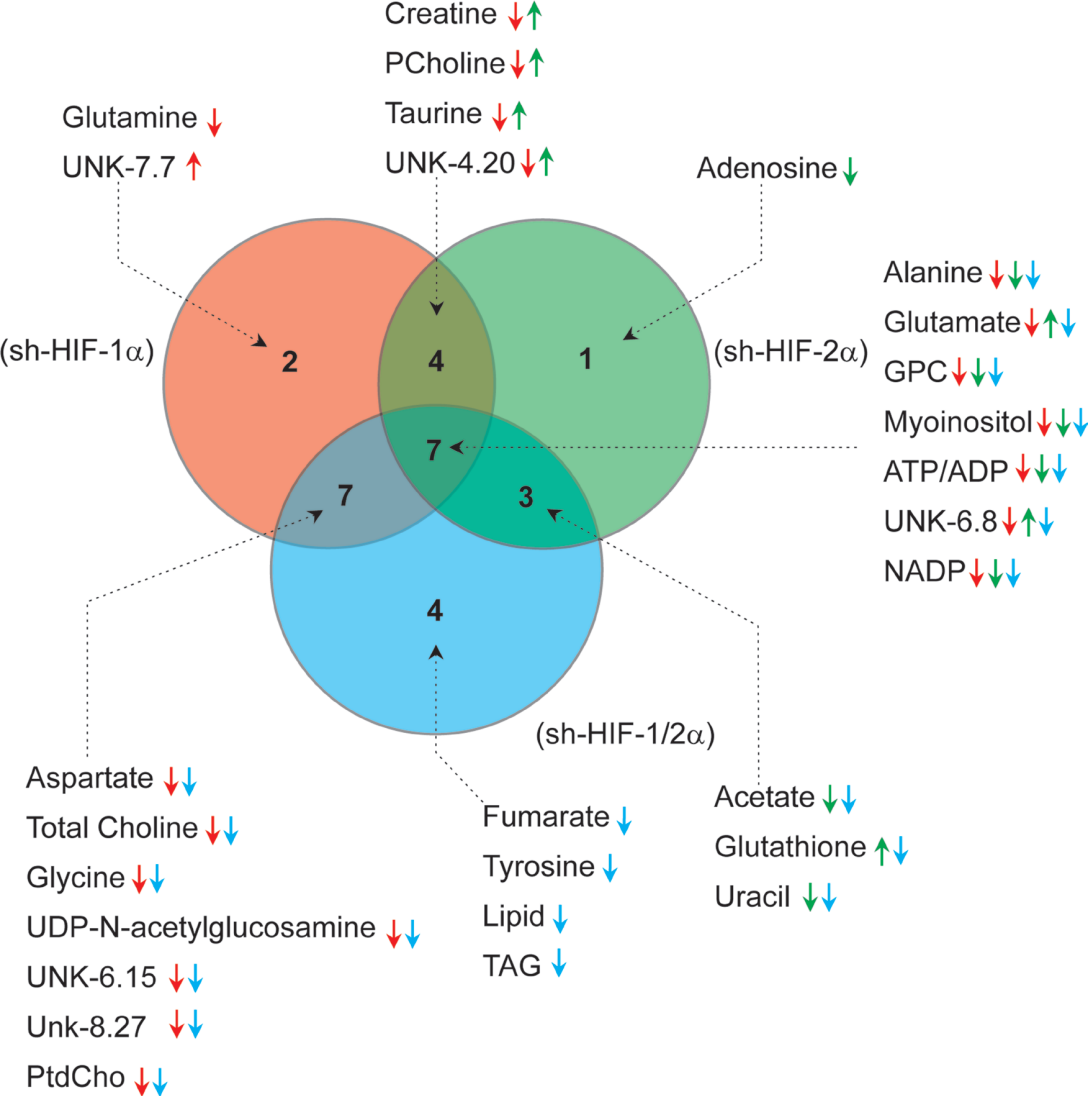
Venn diagram summarizing the commonality and directionality of changes in sh-HIF-1α (red), sh-HIF-2α (green), and both sh-HIF-1/2α (blue) tumors compared to 231-EV tumors, created using web based software Venny 2.1.0 (BioinfoGP at Spanish National Biotechnology Centre (CNB)-CSIC) Numbers of metabolites highlighted in each circle were specific to the respective silenced group. Metabolites that changed in both groups are highlighted in the corresponding overlapping region. The metabolites are listed together with arrows to represent significant increase (up) or decrease (down) in the corresponding groups (red: 231-EV *vs* sh-HIF-1α; green: 231-EV *vs* sh-HIF-2α; blue: 231-EV *vs* sh-HIF-1/2α).

Corresponding bar plots (Figure 5A–5F) demonstrate the significant differences in spectral signals (relative concentration in A.U.) detected in amino acids, organic acids, choline metabolites, substrates/other, and nucleotides in 231-EV, sh-HIF-1α, sh-HIF-2α and sh-HIF-1/2α tumors. Interestingly, changes in metabolites levels were not consistently in the same direction with HIF-1α, HIF-2α, and combined HIF-1α and HIF-2α silencing. The commonality and directionality of the changes are displayed in the Venn diagram in Figure 6.

Glutamine significantly decreased only in HIF-1α silenced tumors. Adenosine significantly decreased but only in HIF-2α silenced tumors. Fumarate and tyrosine decreased significantly but only in tumors with both HIF-1α and HIF-2α silenced. These two compounds decreased with HIF-1α or HIF-2α silencing alone but the decrease was significant only when both HIFs were silenced.

Changes in four metabolites (creatine, phosphocholine, taurine and UNK-4.2 ppm (UNK: unidentified peak)) were common between HIF-1α and HIF-2α silencing. All four metabolites significantly decreased with HIF-1α silencing but significantly increased with HIF-2α silencing, explaining why there was no net change of these metabolites when both HIF-1α and HIF-2α were silenced.

Changes in six metabolites (aspartate, total choline, glycine, UDP-N-AcGlsn, UNK-6.15 and UNK-8.27) were common across sh-HIF-1α and sh-HIF-1/2α tumors and all six significantly decreased suggesting that, except for total choline, HIF-1α silencing but not HIF-2α silencing played a major role in these changes.

Changes in three metabolites (acetate, uracil and glutathione) were common across sh-HIF-2α and sh-HIF-1/2α tumors. Acetate and uracil decreased significantly in both tumor types suggesting that sh-HIF-2α was dominant in this decrease. Glutathione, however, increased significantly with HIF-2α silencing but decreased significantly with combined HIF-1α and HIF-2α silencing although HIF-1α silencing did not significantly decrease this metabolite.

Changes in eight metabolites (alanine, glutamine, glycerophosphocholine, myoinositol, ATP/ADP, UNK-6.8 and ADP) were common to all three tumor types. Of these, alanine, glycerophosphocholine, myoinositol, ATP/ADP and NADP decreased significantly across all three tumor types. The significant decrease of glycerophosphocholine in all three tumor types may explain why total choline decreased in sh-HIF-1α and combined sh-HIF-1/2α tumors despite a significant increase of phosphocholine in sh-HIF-2α tumors. Glutamate decreased significantly with HIF-1α and combined HIF-1α and HIF-2α silencing, but increased significantly with HIF-2α silencing. A similar trend was observed with UNK-6.8 peak.

**Figure 7:**
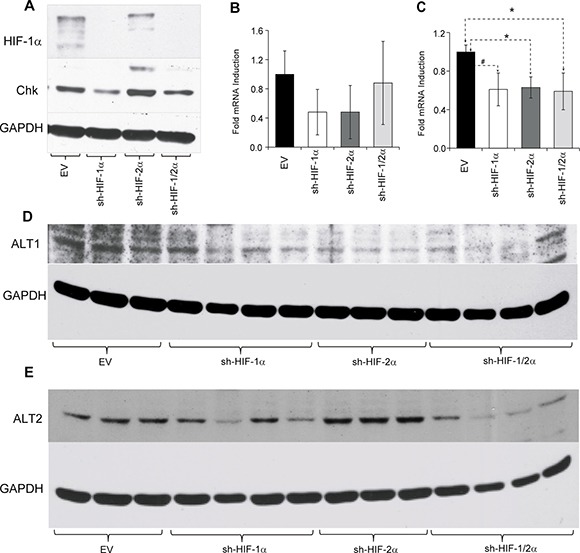
(**A**) Representative western blots showing choline kinase (Chk) expression in tumors derived from empty vector and HIF-silenced cells. Bar plots represents (**B**) alanine aminotransferase 1 (ALT1) and (**C**) alanine aminotransferase 2 (ALT2) mRNA expression levels in tumor xenografts. Values represent mean fold induction ± SEM of the ∆Ct values from 231-EV (*n* = 9), sh-HIF-1α (*n* = 10), sh-HIF-2α (*n* = 8) and sh-HIF-1/2α (*n* = 6) tumors. (**D**) and (**E**) Western blots of ALT1 and ALT2 expression in tumors derived from empty vector and HIF-silenced cells. GAPDH was used as a loading control in the western blots. ^#^ *p* = 0.08; *p* < 0.05.

To further understand the molecular mechanisms underlying some of the metabolic changes we analyzed the protein expression level of choline kinase (Chk) in the tumors (Figure 7A), as well as alanine aminotransferase (ALT) 1 and 2 (Figure 7B–7E). We focused on Chk because of the changes observed in the tumors *in vivo*. Alanine was one of the metabolites that was altered across the sh-HIF-1a, sh-HIF-2a and sh-HIF-1/2a tumors. Silencing of HIF-1α resulted in a decrease of Chk expression, consistent with the significant decrease of phosphocholine observed in these tumors. In contrast, silencing HIF-2α resulted in a significant increase of Chk expression that was consistent with the significant increase of phosphocholine detected in HIF-2α silenced tumors. As a result of these opposing effects, Chk expression and phosphocholine levels were unchanged in the combined HIF-1/2α silenced tumors. Although we did not observe any difference in ALT1 mRNA expression levels (Figure 7B), ALT2 mRNA was significantly lower in all three tumor types (Figure 7C). A decrease of ALT1 protein was observed in sh-HIF-1α, sh-HIF-2α and sh-HIF-1/2α tumors as shown in Figure 7D. ALT2 protein levels decreased in sh-HIF-1α and sh-HIF-1/2α tumors (Figure 7E). The changes in mRNA and protein expression are consistent with the significant decrease of alanine observed in tumors derived from all three sublines.

## DISCUSSION

The occurrence of tumor hypoxia with tumor growth [16] and the presence of hypoxia in human tumors are well established [17]. Since HIFs mediate the adaptive response of cells to hypoxia, silencing HIFs in this TNBC xenograft significantly affected tumor growth only *in vivo*, since cell doubling time under well oxygenated culture conditions was not affected [15]. Silencing HIF-1α, HIF-2α or combined HIF-1α and HIF-2α had a profound effect on tumor growth. Here, for the first time, we identified new metabolic targets of HIF, and also demonstrated the divergent consequences of silencing HIF-1α and HIF-2α individually on some of these targets (summarized in Supplementary Table 1). Multivariate principal component analysis (PCA) of these metabolites showed clear clustering of the different groups (Supplementary Figure 1). The *in vivo* data highlight the spatial metabolic heterogeneity that exists in tumors, and the importance of characterizing tumors *in vivo* in addition to cells to understand the complexities of changes following HIF silencing.

In this TNBC xenograft, combined silencing of both HIF-1α and HIF-2α resulted in a significant decrease of amino acids such as alanine (also decreased with HIF-1α or HIF-2α silencing), aspartate (decreased with HIF-1α but increased with HIF-2α silencing), glutamate (decreased with HIF-1α but increased with HIF-2α silencing), glycine (decreased with HIF-1α but not with HIF-2α silencing) and tyrosine (only decreased with combined HIF-1α and HIF-2α silencing), the organic acids such as acetate (did not change with HIF-1α but decreased with HIF-2α silencing), fumarate (did not change significantly with either HIF-1α or HIF-2α silencing), and pyruvate (did not change significantly with either HIF-1α or HIF-2α silencing), the membrane breakdown product glycerophosphocholine (also decreased with HIF-1α or HIF-2α silencing), the substrates glutathione (did not change with HIF-1α but increased with HIF-2α silencing) and myoinositol (also decreased with HIF-1α or HIF-2α silencing). In addition the nucleotide uracil (also decreased with HIF-2α silencing) decreased with combined HIF-1α and HIF-2α silencing. In the lipid phase extracts, we observed a significant decrease of the lipid signals in combined HIF-1/2α silenced tumors, consistent with the *in vivo* data. We have previously identified changes in lipid droplet formation as a major cause of the reduction of lipids in sh-HIF-2α and sh-HIF-1/2α cells [15]. Unlike cells in culture, *in vivo*, tumor necrosis may also contribute to the lipid signal [18]. The reduction of these compounds in tumors with combined HIF-1α and HIF-2α silenced, together with the profound delay in tumor initiation and reduction in tumor growth from these cells, support their investigation as novel targets in TNBC. In addition, the significant decrease of ATP/ADP and NADP in tumors derived from all three sublines suggests that silencing HIF-1α or HIF-2α or combined HIF-1α and HIF-2α had a profound effect on energy levels and on the pentose phosphate pathway [19, 20].

Elevated alanine, glutamate, and glycine levels have been frequently observed in breast and prostate cancers [21, 22] and have been identified as prognostic markers in TNBC [21]. Alanine and glutamate, are products of glucose and glutamine consumption, and their metabolism in tumors is synthesized by aminotransferase activity [23] that is critical for anchorage independent growth [24]. Cancer cells rapidly convert glutamine to glutamate due to the high expression of mitochondrial glutaminase (GLS).

Glutamate is metabolized to α-ketoglutarate through glutamate dehydrogenase and enters the TCA cycle for the production of pyruvate and ATP. Hypoxia was found to upregulate glutamate dehydrogenase (GDH) in a recent study with lung cancer cells [25]. However we did not detect a decrease of α-ketoglutarate with HIF silencing in our studies, suggesting that glutaminase rather than GDH may have decreased with HIF silencing. Increased secretion of glutamate that disrupts bone homeostasis has been reported in breast and prostate cancer bone metastasis and is responsible, in part, for cancer induced bone pain [26]. Glutamate inhibitors are being developed for the purpose of targeting cancer induced bone pain [27]. Despite a significant decrease of alanine and ALT1/2 expression, glutamine levels increased with HIF-1α silencing and remained unchanged with HIF-2α and combined HIF-1α and HIF-2α silencing.

Apart from glutamine, that is an essential amino acid for cell survival, growth and proliferation, the other major sources of nitrogen carrier/supplier for nucleotide biosynthesis are glycine and aspartate [23]. Glycine, an essential precursor for *de novo* purine nucleotide synthesis significantly decreased in HIF-1α silenced and combined HIF-1α+2α silenced tumors consistent with a previous cell study using Von Hippel-Lindau (VHL) modified human clear cell renal cell carcinoma cells, with HIF-1α, HIF-2α and combined HIF-1α and HIF-2α downregulated [13]. Aspartate contributes to the synthesis of protein and nucleotides and to the electron transfer reaction. Aspartate significantly decreased in HIF-1α silenced and combined HIF-1α and HIF-2α silenced tumors, but increased in HIF-2α silenced tumors.

Creatine, glutathione, taurine and myoinositol decreased with HIF-1α silencing, but with the exception of myoinositol, increased with HIF-2α silencing. Because of these opposing effects, only glutathione and myoinositol decreased significantly in tumors with combined HIF-1α and HIF-2α silenced. Myoinositol is a precursor for the phosphatidylinositol cycle and acts as an osmoregulator at different stages of malignant transformation [28]. Likewise, taurine plays an important role in osmoregulation and volume regulations [29]. Higher concentrations of taurine have been reported in several cancerous tissues, including breast cancer, as compared to non-involved or adjacent tissue [30–32]. Glutathione is an intracellular oxidant and a scavenger of reactive oxygen species [33]. The pattern of changes in glutathione matched the pattern observed with glutamate, a major substrate in glutathione synthesis [34]. The reduction of creatine may reflect an imbalance of energy production through ATP [35].

Increased phosphocholine arising from higher Chk expression is frequently observed in breast and most other cancers, and is associated with increased aggressiveness [36, 37]. An association between Chk and hypoxia has been previously reported [38]. Phosphocholine and Chk decreased in HIF-1α silenced tumors but increased with HIF-2α silencing. As a result phosphocholine did not significantly change with combined HIF-1α and HIF-2α silencing. The increases of Chk and phosphocholine with HIF-2α silencing also suggest that while downregulating HIFs has a dramatic effect on tumor growth, it may also increase aggressiveness within the remaining cells, although this would be dependent upon the net outcome of divergent influences. Phosphocholine is a phospholipid membrane precursor in the formation of phosphatidylcholine, a major component of cell membrane phospholipids. Interestingly, phosphatidylcholine decreased significantly not only in HIF-1α silenced tumors as expected from the decrease of phosphocholine, but also in tumors with combined HIF-1α and HIF-2α silencing. Glycerophosphocholine is a phospholipid membrane breakdown product that decreased in tumors with HIF-1α, HIF-2α and combined HIF-1α and HIF-2α silencing, resulting in an overall decrease of total choline that was also confirmed *in vivo*. Phosphatidylethanolamine that is formed from phosphoethanolamine was not significantly affected suggesting that this pathway was not influenced by hypoxia and HIFs in these tumors.

Acetate, fumarate and pyruvate significantly decreased in tumors with combined HIF-1α and HIF-2α silencing. Only fumarate decreased with HIF-2α silencing, but all three were not significantly altered with HIF-1α silencing. Interestingly, unlike results from cell studies that report on decreased glucose consumption and reduced lactate production [13, 15], in tumors we did not observe a significant change in glucose or lactate levels with HIF-1α, HIF-2α or both HIF-1α and HIF-2α silenced. In tumors, vascularization driven substrate delivery plays a major role in the formation of metabolites. Silencing of HIFs may have decreased vascularization and substrate delivery that may explain why the changes in glucose consumption and lactate production observed in cells were not observed *in vivo*. Although tumor tissue was snap frozen in liquid nitrogen within seconds, lactate quantification from extracts may not be completely reliable and lactate was not quantified *in vivo*. Tumor vasculature is typically heterogeneous [39] that may explain the spatial heterogeneity of the total choline maps *in vivo*. In addition, our studies were performed with immune compromised mice to allow growth of human TNBC lines. Future studies with humanized mice with intact immune systems should provide insights into how the HIF regulated pathways and the resultant metabolic changes observed here, especially changes observed in glutamine and glutamate, may modify immune environments in tumors [40, 41]. Comparisons in our studies were made with tumors derived from empty vector transfected cells, although ideally additional control tumors should have included tumors derived from MDA-MB-231 cells expressing scrambled shRNA.

Previous studies performed with HIF-1α deficient HCT 116 human colorectal and murine hepatoma HEPA-1 cancer cells [14], as well as with VHL modified human clear cell renal cell carcinoma cells with HIF-1α, HIF-2α and combined HIF-1α and HIF-2α downregulation [13] have provided a wealth of information on the effects of HIF downregulation on cell metabolism. Although cells in culture do not have the complex microenvironment of tumors *in vivo*, several changes observed in these cells were replicated in our observations with MDA-MB-231 tumors. In addition, for the first time we observed changes in fumarate, uracil, nucleosides, lipids and phosphatidylcholine in tumors with HIF silencing.

Our data illustrate the metabolic complexities of the HIF pathways, and the importance of silencing both isoforms of HIF to disrupt metabolic adaptation. In several instances, compounds that decreased with HIF-1α silencing increased with HIF-2α silencing suggesting that the two pathways may have compensatory roles. Understanding the role of HIF-α isoforms in cancer metabolism may provide new insights into targeting hypoxia and improving response to chemo, radiation, and immune therapy.

## MATERIALS AND METHODS

### Cells and cell culture conditions

Cloning and generation of MDA-MB-231 cells stably expressing shRNA against HIF-1α (sh-HIF-1α), HIF-2α (sh-HIF-2α) and both HIF-1α and HIF-2α (sh-HIF-1/2α) using lentiviral transduction is detailed in previously published papers [15, 42]. The three genetically engineered MDA-MB-231 sublines sh-HIF-1α, sh-HIF-2α and sh-HIF1/2α, and empty vector MDA-MB-231 cells with no shRNA (231-EV) were maintained in RPMI 1640 medium (Mediatech, Manassas, VA, USA) supplemented with 10% fetal bovine serum (Sigma, St. Louis, MO, USA), and maintained at 37°C in a CO_2_ incubator.

### Establishment of xenografts

Tumors were generated by injecting 2 × 10^6^ 231-EV, sh-HIF-1α, sh-HIF-2α, or sh-HIF-1/2α cells in the mammary fat pad of severe combined immunodeficient (SCID) female mice. Weekly caliper measurements of tumor volumes were used to evaluate tumor growth. Number of mice investigated in each group were *n* = 9 for 231-EV, *n* = 10 for sh-HIF-1α, *n* = 9 for sh-HIF-2α and *n* = 7 for 231-HIF-1/2α unless otherwise stated. At the end of the MR imaging studies, tumors were harvested, and partly fixed in formalin for histology and partly processed for molecular and metabolic studies. All surgical procedures and animal handling were performed in compliance with guidelines and protocols approved by the Johns Hopkins University Institutional Animal Care and Use Committee, and conformed to the Guide for the Care and Use of Laboratory Animals published by the NIH.

### *In vivo* ^1^H MR spectroscopic imaging

Once tumor volumes were ∼250mm^3^, mice were anesthetized and imaged on a 9.4T Bruker Biospec spectrometer (Bruker Biospin Corp. Billerica, MA, USA). Body temperature of the animals was maintained during imaging by a thermostat-regulated heating pad. Localized *in vivo* proton spectra from tumors were acquired using a homebuilt solenoid coil placed around the tumor. Spectra from a 4 mm thick slice were acquired with a field of view of 16 mm; a matrix size of 16 × 16 × 1024; 4 scans per phase encode step; an echo time (TE) of 120 milliseconds, and a repetition time (TR) of 1 second, using a 2D-CSI (chemical shift imaging) sequence with VAPOR water suppression [43].

Signals at approximately 3.2 ppm from total choline (sum of choline, phosphocholine, and glycerophosphocholine) and at 1.3 ppm from lipids with some contribution from lactate that overlaps with the lipid signal were detected in localized proton MR spectra. Reference 2D-CSI images with unsuppressed water signal were acquired with TE = 20 milliseconds and number of scan (NS) = 1, keeping all other parameters the same. Quantitative maps of total choline and lipids were generated from the spectroscopic images using unsuppressed water signal as an internal reference using an in-house IDL program [38, 44].

### mRNA and protein expression

Total RNA was isolated from snap frozen tumors using QIAshredder and RNeasy Mini kit (Qiagen, Valencia, CA, USA) as per the manufacturer’s protocol. cDNA was prepared using the iScript cDNA synthesis kit (Bio-Rad, Hercules, CA, USA). cDNA samples were diluted 1:10 and real-time PCR was performed using IQ SYBR Green supermix and gene specific primers in the iCycler real-time PCR detection system (Bio-Rad). All primers were designed using Beacon designer software 7.8 (premier Biosoft, Palo Alto, CA, USA). The expression of target RNA relative to the housekeeping gene HPRT1 was calculated based on the threshold cycle (Ct) as R = 2-Δ(ΔCt), where ΔCt = Ct of target - Ct of HPRT1.

Expression levels of HIF-1α, HIF-2α, Chk, ALT1 and ALT2 were obtained by immunoblotting. GAPDH was used as a loading control. After blocking with 5% nonfat milk, samples were incubated overnight with mouse monoclonal anti-HIF-1α antibody (1:500 dilution; BD Bioscience, San Jose, CA) or rabbit polyclonal anti-HIF-2α antibody (1:500; GeneTex, Inc. Irvine, CA, USA), or human-specific rabbit polyclonal Chk (1:100 dilution, custom made), or rabbit polyclonal ALT1 (1:1000, Proteintech, Rosemont, IL, USA), or rabbit polyclonal ALT2 (Proteintech) or monoclonal anti-GAPDH (1:50,000, SIGMA). Horseradish peroxidase-conjugated secondary antibodies were used at 1:2000 dilution. Blots were visualized using the SuperSignal West Pico Chemiluminescent substrate kit (Thermo Scientific, Rockford, IL, USA).

### Dual-phase extraction and high-resolution ^1^H MRS analysis

Water-soluble and lipid fractions were extracted from tumor tissue using a dual-phase extraction method [45]. Briefly, the homogenized tumor was mixed with 4 mL of ice-cold methanol and vigorously vortexed. After keeping samples on ice for 10 minutes, 4 mL of chloroform were added, vortexed vigorously and kept on ice for an additional 10 minutes. Ultrasonication under ice-cold conditions was performed for 5 minute with a 1-second pulse interval to make sure all the tissues dissolved completely. Finally, 4 mL of water were added and the samples were vortexed again. All procedures were performed on ice and samples were stored at 4°C overnight for phase separation and then centrifuged at 15,000 × g at 4°C for 10 minutes. The aqueous phase containing water-soluble metabolites was collected [43]. Methanol in the aqueous phase was first evaporated under nitrogen gas, and any water remaining in the aqueous phase was lyophilized. Dried aqueous phase extracts were re-suspended in 0.6 mL deuterated water (D_2_O) for MRS analysis. TSP (3-(trimethylsilyl) propionic 2,2,3,3-d4 acid sodium salt) dissolved in D_2_O was used as an internal standard. Lipid phase extracts were dried under nitrogen gas stream and re-suspended in 0.6 mL deuterated chloroform and methanol in a 2:1 ratio containing TMS 0.05% v/v.

Fully relaxed ^1^H MR spectra of aqueous and lipid phase extracts were acquired on a Bruker Avance 500 spectrometer (Bruker BioSpin Corp.) as previously described [36]. For quantitative analysis of metabolites, integrals of resonances were determined and normalized to tumor weight and compared to the TSP standard (aqueous phase) or TMS standard (lipid phase) to obtain relative concentrations in arbitrary units (A.U.).

### Statistical analysis

To determine the statistical significance of the quantified data, an unpaired one tailed Student’s *T*-test was performed using Microsoft Office Excel 2010 (Microsoft, Redmond, WA, USA). *P* values ≤ 0.05 were considered significant unless otherwise stated.

## SUPPLEMENTARY FIGURE AND TABLE



## Abbreviations

HIF-1α: Hypoxia Inducible Factor 1alpha
HIF-2α: Hypoxia Inducible Factor 2alpha
Chk: Choline Kinase
MRS: Magnetic Resonance Spectroscopy
ATP/ADP: Adenosine triphosphate/Adenosine diphosphate
NADP: Nicotinamide adenine dinucleotide phosphate.

## References

[1] Vaupel P (2008). Hypoxia and aggressive tumor phenotype: implications for therapy and prognosis. Oncologist.

[2] Vaupel P, Thews O, Hoeckel M (2001). Treatment resistance of solid tumors: role of hypoxia and anemia. Med Oncol.

[3] Gilkes DM, Semenza GL (2013). Role of hypoxia-inducible factors in breast cancer metastasis. Future Oncol.

[4] Penet MF, Chen Z, Bhujwalla ZM (2011). MRI of metastasis-permissive microenvironments. Future Oncol.

[5] Semenza GL (2007). Hypoxia-inducible factor 1 (HIF-1) pathway. Sci STKE.

[6] Hu CJ, Sataur A, Wang L, Chen H, Simon MC (2007). The N-terminal transactivation domain confers target gene specificity of hypoxia-inducible factors HIF-1alpha and HIF-2alpha. Mol Biol Cell.

[7] Tian H, McKnight SL, Russell DW (1997). Endothelial PAS domain protein 1 (EPAS1), a transcription factor selectively expressed in endothelial cells. Genes Dev.

[8] Semenza GL (2010). Defining the role of hypoxia-inducible factor 1 in cancer biology and therapeutics. Oncogene.

[9] Keith B, Johnson RS, Simon MC (2011). HIF1α and HIF2α: sibling rivalry in hypoxic tumour growth and progression. Nat Rev Cancer.

[10] Dent R, Trudeau M, Pritchard KI, Hanna WM, Kahn HK, Sawka CA, Lickley LA, Rawlinson E, Sun P, Narod SA (2007). Triple-negative breast cancer: clinical features and patterns of recurrence. Clin Cancer Res.

[11] Gangi A, Chung A, Mirocha J, Liou DZ, Leong T, Giuliano AE (2014). Breast-conserving therapy for triple-negative breast cancer. JAMA Surg.

[12] Jin MS, Lee H, Park IA, Chung YR, Im SA, Lee KH, Moon HG, Han W, Kim K, Kim TY, Noh DY, Ryu HS (2016). Overexpression of HIF1α and CAXI predicts poor outcome in early-stage triple negative breast cancer. Virchows Arch.

[13] Cuperlovic-Culf M, Cormier K, Touaibia M, Reyjal J, Robichaud S, Belbraouet M, Turcotte S (2016). (1)H NMR metabolomics analysis of renal cell carcinoma cells: effect of VHL inactivation on metabolism. Int J Cancer.

[14] Armitage EG, Kotze HL, Allwood JW, Dunn WB, Goodacre R, Williams KJ (2015). Metabolic profiling reveals potential metabolic markers associated with Hypoxia Inducible Factor-mediated signalling in hypoxic cancer cells. Nature.

[15] Shah T, Krishnamachary B, Wildes F, Mironchik Y, Kakkad SM, Jacob D, Artemov D, Bhujwalla ZM (2015). HIF isoforms have divergent effects on invasion, metastasis, metabolism and formation of lipid droplets. Oncotarget.

[16] Büchler P, Reber HA, Lavey RS, Tomlinson J, Büchler MW, Friess H, Hines OJ (2004). Tumor hypoxia correlates with metastatic tumor growth of pancreatic cancer in an orthotopic murine model1. J Surg Res.

[17] Dales JP, Garcia S, Meunier-Carpentier S, Andrac-Meyer L, Haddad O, Lavaut MN, Allasia C, Bonnier P, Charpin C (2005). Overexpression of hypoxia-inducible factor HIF-1α predicts early relapse in breast cancer: retrospective study in a series of 745 patients. Int J Cancer.

[18] Zoula S, Hérigault G, Ziegler A, Farion R, Décorps M, Rémy C (2003). Correlation between the occurrence of 1H-MRS lipid signal, necrosis and lipid droplets during C6 rat glioma development. NMR Biomed.

[19] Yamashita A, Zhao Y, Matsuura Y, Yamasaki K, Moriguchi-Goto S, Sugita C, Iwakiri T, Okuyama N, Koshimoto C, Kawai K, Tamaki N, Zhao S, Kuge Y, Asada Y (2014). Increased metabolite levels of glycolysis and pentose phosphate pathway in rabbit atherosclerotic arteries and hypoxic macrophage. PLoS One.

[20] Semenza GL (2017). Hypoxia-inducible factors: coupling glucose metabolism and redox regulation with induction of the breast cancer stem cell phenotype. EMBO J.

[21] Cao MD, Lamichhane S, Lundgren S, Bofin A, Fjosne H, Giskeodegard GF, Bathen TF (2014). Metabolic characterization of triple negative breast cancer. BMC Cancer.

[22] Tessem MB, Swanson MG, Keshari KR, Albers MJ, Joun D, Tabatabai ZL, Simko JP, Shinohara K, Nelson SJ, Vigneron DB, Gribbestad IS, Kurhanewicz J (2008). Evaluation of lactate and alanine as metabolic biomarkers of prostate cancer using 1H HR-MAS spectroscopy of biopsy tissues. Magn Reson Med.

[23] DeBerardinis RJ, Cheng T (2010). Q’s next: the diverse functions of glutamine in metabolism, cell biology and cancer. Oncogene.

[24] Weinberg F, Hamanaka R, Wheaton WW, Weinberg S, Joseph J, Lopez M, Kalyanaraman B, Mutlu GM, Budinger GR, Chandel NS (2010). Mitochondrial metabolism and ROS generation are essential for Kras-mediated tumorigenicity. Proc Natl Acad Sci USA.

[25] Jiang ZF, Wang M, Xu JL, Ning YJ (2017). Hypoxia promotes mitochondrial glutamine metabolism through HIF1alpha-GDH pathway in human lung cancer cells. Biochem Biophys Res Commun.

[26] Seidlitz EP, Sharma MK, Singh G (2010). Extracellular glutamate alters mature osteoclast and osteoblast functions. Can J Physiol Pharmacol.

[27] Fazzari J, Lin H, Murphy C, Ungard R, Singh G (2015). Inhibitors of glutamate release from breast cancer cells; new targets for cancer-induced bone-pain. Sci Rep.

[28] Wang H, Wang L, Zhang H, Deng P, Chen J, Zhou B, Hu J, Zou J, Lu W, Xiang P, Wu T, Shao X, Li Y (2013). ¹H NMR-based metabolic profiling of human rectal cancer tissue. Mol Cancer.

[29] Griffin JL, Shockcor JP (2004). Metabolic profiles of cancer cells. Nat Rev Cancer.

[30] Sitter B, Lundgren S, Bathen TF, Halgunset J, Fjosne HE, Gribbestad IS (2006). Comparison of HR MAS MR spectroscopic profiles of breast cancer tissue with clinical parameters. NMR Biomed.

[31] Bathen TF, Geurts B, Sitter B, Fjosne HE, Lundgren S, Buydens LM, Gribbestad IS, Postma G, Giskeodegard GF (2013). Feasibility of MR metabolomics for immediate analysis of resection margins during breast cancer surgery. PLoS One.

[32] Beckonert O, Monnerjahn J, Bonk U, Leibfritz D (2003). Visualizing metabolic changes in breast-cancer tissue using 1H-NMR spectroscopy and self-organizing maps. NMR Biomed.

[33] Birben E, Sahiner UM, Sackesen C, Erzurum S, Kalayci O (2012). Oxidative stress and antioxidant defense. World Allergy Organ J.

[34] Lu SC (2009). Regulation of glutathione synthesis. Mol Aspects Med.

[35] Schlattner U, Tokarska-Schlattner M, Wallimann T (2006). Mitochondrial creatine kinase in human health and disease. Biochimica et Biophysica Acta (BBA) - Molecular Basis of Disease.

[36] Penet MF, Shah T, Bharti S, Krishnamachary B, Artemov D, Mironchik Y, Wildes F, Maitra A, Bhujwalla ZM (2015). Metabolic imaging of pancreatic ductal adenocarcinoma detects altered choline metabolism. Clin Cancer Res.

[37] Bhujwalla ZM, Aboagye EO, Gillies RJ, Chacko VP, Mendola CE, Backer JM (1999). Nm23-transfected MDA-MB-435 human breast carcinoma cells form tumors with altered phospholipid metabolism and pH: a 31P nuclear magnetic resonance study in vivo and in vitro. Magn Reson Med.

[38] Glunde K, Shah T, Winnard PT, Raman V, Takagi T, Vesuna F, Artemov D, Bhujwalla ZM (2008). Hypoxia regulates choline kinase expression through hypoxia-inducible factor-1 alpha signaling in a human prostate cancer model. Cancer Res.

[39] Raman V, Artemov D, Pathak AP, Winnard PT, McNutt S, Yudina A, Bogdanov A, Bhujwalla ZM (2006). Characterizing Vascular Parameters in Hypoxic Regions: A Combined Magnetic Resonance and Optical Imaging Study of a Human Prostate Cancer Model. Cancer Res.

[40] Knox WE, Tremblay GC, Spanier BB, Friedell GH (1967). Glutaminase activities in normal and neoplastic tissues of the rat. Cancer Res.

[41] Hammami I, Jolicoeur M, Rajendram R, Preedy VR, Patel VB (2015). Glutamine and Cancer Immunosuppression. Glutamine in Clinical Nutrition.

[42] Krishnamachary B, Penet MF, Nimmagadda S, Mironchik Y, Raman V, Solaiyappan M, Semenza GL, Pomper MG, Bhujwalla ZM (2012). Hypoxia regulates CD44 and its variant isoforms through HIF-1α in triple negative breast cancer. PLoS One.

[43] Tkac I, Starcuk Z, Choi IY, Gruetter R (1999). In vivo 1H NMR spectroscopy of rat brain at 1 ms echo time. Magn Reson Med.

[44] Bolan PJ, Meisamy S, Baker EH, Lin J, Emory T, Nelson M, Everson LI, Yee D, Garwood M (2003). In vivo quantification of choline compounds in the breast with 1H MR spectroscopy. Magn Reson Med.

[45] Glunde K, Raman V, Mori N, Bhujwalla ZM (2005). RNA interference-mediated choline kinase suppression in breast cancer cells induces differentiation and reduces proliferation. Cancer Res.

