# Supplementary material for: Metabolic consequences of HIF silencing in a triple negative human breast cancer xenograft
Source: Oncotarget. 2018 Feb 24;9(20):15326–39. doi: 10.18632/oncotarget.24569 (PMC5880607; doi:10.18632/oncotarget.24569)
Supplement: Supplementary file 1 [file oncotarget-09-15326-s001.pdf]

Metabolic consequences of HIF silencing in a triple negative human breast cancer xenograft

SUPPLEMENTARY MATERIALS

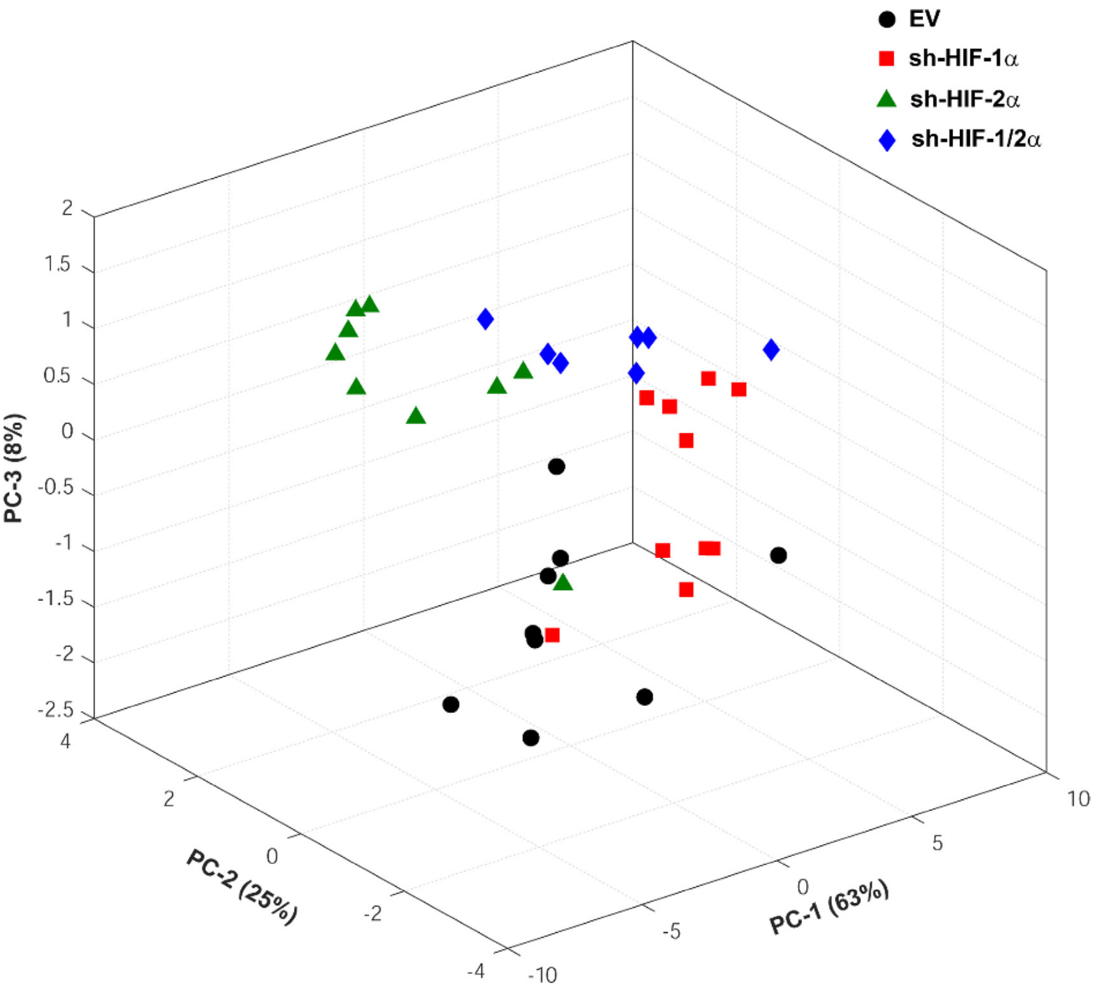

Supplementary Figure 1: Multivariate principal component analysis (PCA) of metabolites. A three dimensional scatter score plot shows clear clustering of 231-EV (black dot), sh-HIF-1α (red square), sh-HIF-2α (green triangle), and sh-HIF-1/2α (blue diamond) tumors.

Supplementary Table 1: Metabolites identified and quantified in aqueous and lipid phase extracts of tumors derived from EV, sh-HIF-1α, sh-HIF-2α shRNA, sh-HIF-1/2α cells

	**Metabolites**	**Chem Shift**	**EV**		**sh-HIF-1α**			**sh-HIF-2α**			**sh-HIF-1/2α**		
		**ppm**	**Avg**	**± SEM**	**Avg**	**± SEM**	**[*a]*P*-value**	**Avg**	**± SEM**	**[# b]*P*-value**	**Avg**	**± SEM**	**[c]*P*-value**
**Amino Acids**	BCA	0.99	1.01	± 0.10	1.03	± 0.111	(0.436)	0.96	± 0.068	(0.343)	0.88	± 0.119	(0.210)
	Valine	1.05	0.17	± 0.018	0.19	± 0.02	(0.212)	0.17	± 0.012	(0.458)	0.16	± 0.023	(0.348)
	Alanine	1.48	1.21	± 0.082	0.78	± 0.09	(0.002)	0.77	± 0.056	(0.001)	0.63	± 0.086	(0.000)
	Lysine	1.73	0.84	± 0.05	0.76	± 0.079	(0.210)	0.84	± 0.06	(0.467)	0.73	± 0.10	(0.153)
	Glutamate	2.35	1.84	± 0.096	1.29	± 0.16	(0.005)	2.15	± 0.15	(0.052)	1.16	± 0.135	(0.000)
	Glutamine	2.45	0.53	± 0.054	0.40	± 0.039	(0.033)	0.63	± 0.044	(0.083)	0.44	± 0.043	(0.118)
	Aspartate	2.80	0.16	± 0.014	0.07	± 0.018	(0.000)	0.15	± 0.019	(0.245)	0.05	± 0.018	(0.000)
	Glycine	3.57	0.76	± 0.067	0.40	± 0.046	(0.001)	0.81	± 0.073	(0.317)	0.44	± 0.055	(0.002)
	Tyrosine	6.90	0.04	± 0.005	0.04	± 0.005	(0.185)	0.03	± 0.005	(0.133)	0.03	± 0.003	(0.025)
	Phenylalanine	7.41	0.08	± 0.01	0.09	± 0.009	(0.253)	0.07	± 0.004	(0.239)	0.07	± 0.008	(0.284)
	Histidine	7.69	0.01	± 0.004	0.01	± 0.004	(0.330)	0.007	± 0.002	(0.212)	0.01	± 0.004	(0.321)
**Organic Acids**	Lactate	1.34	6.87	± 0.702	6.23	± 0.719	(0.269)	5.56	± 0.347	(0.057)	6.10	± 0.885	(0.250)
	Acetate	1.92	0.26	± 0.068	0.16	± 0.024	(0.079)	0.13	± 0.019	(0.038)	0.11	± 0.022	(0.042)
	Fumarate	6.51	0.005	± 0.001	0.003	± 0.001	(0.222)	0.004	± 0.001	(0.308)	0.002	± 0.001	(0.052)
	Formate	8.45	0.06	± 0.019	0.056	± 0.009	(0.429)	0.042	± 0.005	(0.193)	0.042	± 0.012	(0.240)
	Pyruvate	2.40	0.33	± 0.034	0.25	± 0.037	(0.067)	0.31	± 0.016	(0.276)	0.23	± 0.024	(0.022)
**Cholines**	Choline	3.21	0.79	± 0.111	0.61	± 0.084	(0.108)	0.83	± 0.069	(0.358)	0.57	± 0.064	(0.073)
	PC	3.22	2.90	± 0.195	1.79	± 0.237	(0.001)	4.77	± 0.51	(0.002)	3.07	± 0.325	(0.325)
	GPC	3.24	3.45	± 0.284	1.97	± 0.318	(0.002)	2.30	± 0.195	(0.002)	1.28	± 0.183	(0.000)
	Total-Choline	3.22	6.81	± 0.469	4.16	± 0.537	(0.001)	7.54	± 0.596	(0.176)	4.67	± 0.506	(0.004)
	Creatine	3.04	0.50	± 0.044	0.39	± 0.032	(0.025)	0.79	± 0.069	(0.001)	0.59	± 0.064	(0.113)
**Substrate / Others**	Glucose	5.24	0.05	± 0.018	0.02	± 0.011	(0.102)	0.07	± 0.024	(0.233)	0.08	± 0.028	(0.153)
	Glutathione	2.54	0.77	± 0.048	0.64	± 0.095	(0.133)	0.99	± 0.066	(0.009)	0.52	± 0.067	(0.004)
	Taurine	3.43	2.83	± 0.17	2.10	± 0.197	(0.007)	4.17	± 0.326	(0.001)	2.44	± 0.359	(0.155)
	Myo-inositol	4.06	0.79	± 0.064	0.27	± 0.038	(0.001)	0.51	± 0.05	(0.002)	0.33	± 0.045	(0.000)
**Nucleotides**	Uracil	5.80	0.009	± 0.002	0.008	± 0.002	(0.966)	0.002	± 0.001	(0.033)	0.001	± 0.001	(0.015)
	Adenosine	8.36	0.06	± 0.007	0.06	± 0.01	(0.395)	0.04	± 0.006	(0.025)	0.04	± 0.009	(0.151)
	UDP-N-AcGlsn+	5.51	0.047	± 0.004	0.015	± 0.007	(0.001)	0.04	± 0.006	(0.183)	0.01	± 0.007	(0.000)
	NADP	9.32	0.03	± 0.003	0.02	± 0.004	(0.003)	0.02	± 0.003	(0.009)	0.01	± 0.002	(0.000)
	ATP/ADP	8.51	0.17	± 0.019	0.05	± 0.013	(0.000)	0.12	± 0.006	(0.015)	0.06	± 0.014	(0.000)
**Unidentified**	UNK-4.20	4.17	0.97	± 0.059	0.67	± 0.08	(0.004)	1.48	± 0.138	(0.002)	0.95	± 0.09	(0.425)
	UNK-6.15	6.15	0.26	± 0.026	0.088	± 0.025	(0.000)	0.26	± 0.022	(0.497)	0.134	± 0.033	(0.003)
	UNK-6.8	6.80	0.016	± 0.001	0.003	± 0.001	(0.000)	0.024	± 0.002	(0.006)	0.01	± 0.002	(0.028)
	UNK-7.70	7.70	0.001	± 0.001	0.04	± 0.003	(0.000)	0.025	± 0.017	(0.08)	0.04	± 0.03	(0.056)
	UNK-8.27	8.27	0.25	± 0.02	0.105	± 0.017	(0.000)	0.241	± 0.017	(0.385)	0.134	± 0.025	(0.002)

Student *t*-test [*]a; EV vs HIF-1α shRNA, [#]b; EV vs HIF-2α shRNA, C; EV vs HIF-1α+2α shRNA, +UDP-N-Acetyl Glucosamine
Values represent Mean ± SEM. A one-tailed student t-test was performed on quantified integral peak areas. *P*-values < 0.05 were considered statistically significant. Peak integral areas were normalized to sample weight and dilution factors used during the experimental procedures.
